# Green symphonies: a call for studies on acoustic communication in plants

**DOI:** 10.1093/beheco/ars206

**Published:** 2012-11-25

**Authors:** Monica Gagliano

**Affiliations:** ^a^Centre for Evolutionary Biology, School of Animal Biology, University of Western Australia, Crawley, Australia and; ^b^Centre for Microscopy, Characterisation and Analysis, University of Western Australia, Crawley WA 6009, Australia

**Keywords:** behavior, bioacoustics, communication, frequencies, plants signaling, sound.

## Abstract

Sound and its use in communication have significantly contributed to shaping the ecology, evolution, behavior, and ultimately the success of many animal species. Yet, the ability to use sound is not a prerogative of animals. Plants may also use sound, but we have been unable to effectively research what the ecological and evolutionary implications might be in a plant’s life. Why should plants emit and receive sound and is there information contained in those sounds? I hypothesize that it would be particularly advantageous for plants to learn about the surrounding environment using sound, as acoustic signals propagate rapidly and with minimal energetic or fitness costs. In fact, both emission and detection of sound may have adaptive value in plants by affecting responses in other organisms, plants, and animals alike. The systematic exploration of the functional, ecological, and evolutionary significance of sound in the life of plants is expected to prompt a reinterpretation of our understanding of these organisms and galvanize the emergence of novel concepts and perspectives on their communicative complexity.

Communication is ubiquitous in nature and is arguably one of the most studied topics in the behavioral sciences. Although the search for a rigorous and comprehensive definition of communication has been and still is at the heart of much debate (Scott-Phillips 2007; Carazo and Font 2010), the basic phenomenon involves the intentional transfer of some kind of information or signal that benefit both the emitter and the receiver. The flow of information between species (or individuals within species) relies on the sender’s encoding mechanisms and the receiver’s decoding mechanisms, and it is the relationship between how information is packaged (encoding) and the content translated (decoding) that determines the outcome of communicative interactions (Wiley 1983). Any such exchange of information between living organisms, irrespective of their level of biological organization, can be considered communication, and as such communication is ubiquitous. Historically, the study of communication processes has primarily focused on animals, probably because their signal-mediated interactions often involve loud and bold displays and eye-catching movements of distinctive body parts, which have clearly succeeded in attracting our attention. The notion of communication in plants has long been regarded as a controversial fringe idea because the exchange of information in plants was thought to involve cues (i.e., incidental features present in the environment that have not been shaped by natural selection to carry a specific meaning for intended receivers and which most researchers agree should not be considered communicative signals; see Bradbury and Vehrencamp 1998; Maynard Smith and Harper 2003) rather than signals (i.e., traits that evolved for a specific role in communication; see definition by Scott-Phillips 2007). Yet this attitude has been rapidly changing as plant communication research attracts increasingly widespread attention (Baldwin and Schultz 1983; Dicke et al. 2003; Baluška et al. 2005; Karban 2008).

Over the last 2 decades, important insights into our understanding of plant ecology, and specifically chemical signaling, have confirmed that plants indeed communicate. An elegant example of this is shown by the relay of a “drought alarm” signal by the garden pea, *Pisum sativum* (Falik et al. 2011). As well as perceiving and responding to the stress cues emitted by drought-stressed neighbors by closing their stomata, unstressed plants signal information of the impending conditions (and elicit stress responses, i.e., reduction of stomatal aperture) to unstressed plants located further away from the stressed plants. Under drought conditions, the decreased water availability causes a reduction in the uptake of nutrients essential to plant growth (resulting in the decline of leaf size, stem extension, and root proliferation; Farooq et al. 2009) and makes plants more palatable to herbivores (e.g., Gutbrodt et al. 2012). Hence, sharing information on imminent drought stress benefits both signaler and receiver plants by enabling them to minimize the direct effect of water deficit on growth, but most importantly, curtail the indirect effect of attracting unwanted visitors in the area at a time of stress, hence minimizing further tissues loss (which represent a large proportion of the reproductive capacity of the plant; reviewed by Chapin 1991). The literature is replete with similar studies demonstrating how plants use chemicals, contact, or various light wavelengths to transmit, receive, and evaluate information about their neighbors both above (Smith 2000) and below ground (Gersani et al. 2001; Gruntman and Novoplansky 2004; Murphy and Dudley 2007), as well as about the resources available in their surroundings, and modify their variable growth and development accordingly (Trewavas 2003; Baluška and Mancuso 2007). For example, plants exchange information to recognize and even prevent costly competitive interactions with relatives by favoring them over strangers (Dudley and File 2007; Murphy and Dudley 2009), and hence facilitating kin selection processes such as cooperation and altruism, similar to what is seen in animal social systems.

Although the proximate and ultimate mechanisms used by animals to sense their environment and communicate with each other have long been the subject of intense scientific interest, the study of plant communication exists, but is still not as advanced and recognized. This is particularly the case for plant bioacoustics; and it is surprising, when we consider that the ability to sense sound and vibrations is a phylogenetically ancient sensory modality behind the behavioral organization of all living organisms and their relationship with their environment (e.g., Jacobs et al. 2007; Müller and Tsuji 2007; Manley and Fuchs 2011).

## A WORLD FULL OF SOUND

From the submicroscopic world of atoms and molecules to the macroscopic world of earthquakes and tsunamis, energy exists everywhere in the form of vibrations and often exhibits a wave-like behavior as it moves throughout space and time (Hewitt 2002). As waves propagate, they transport energy as well as a varying amount of information about everything they encounter, and living organisms have evolved ingenious ways of utilizing wave motion of various kinds as information couriers. Virtually all communication depends on waves of some nature and sound waves offer one of the best examples of this. Specifically, sound waves of many different frequencies and sources constantly travel back and forth through the environment we live in and tell us a great deal about the surrounding world. Certainly, sound has been a source of communication and expression for our species for over 300 000 years (Holden 2004). Generally, our awareness of a sound depends on its loudness, which is strongly correlated with the intensity of the sound and affected by its frequency content (and of course, the density of the medium it travels through). Specifically, the intensity of the signal measures the amplitude of the sound wave (i.e., the amount of energy in the wave) and determines how far that acoustic wave can travel. The frequency of the vibration (i.e., how tightly wave peaks are packed in time) measures the “pitch” of the acoustic signal, determining whether the sound will be heard at all (but note that many natural sounds are complex and span a wide range of frequencies). Because the perception of sound in humans is limited to audio frequencies in the range of 20–20 000 Hz, species that exploit acoustic frequencies outside the pitch of human ears appear silent to us. Nonetheless, from the very low infrasonic (<20 Hz) long-distance calls of the African elephant (Langbauer et al. 1991; Garstang 2004) to the ultrasonic (>20kHz) “conversations” of bacteria (Matsuhashi et al. 1998), the very high pitched vocalizations of many rodents, bats (Arch and Narins 2008), and some singing frogs (Feng et al. 2006), we now know that many of them are clearly quite noisy.

Bioacoustics is the branch of science concerned with sounds produced by or affecting living organisms, especially as relating to communication. Traditionally, bioacoustics aims at recording and studying the sounds that different animal species produce within the context of the natural environment in which they live. Whether such selective focus on animals as the main sound source in an environment is cognitively and/or socially ingrained (Schussler and Olzak 2008), it is undoubtedly limiting. In fact, there is a type of sound-producing biotic component that exists in almost every environment and that is customarily overlooked: plants (Lopez 2004). Besides the audible sounds from plant leaves and branches as raindrops touch them or the wind sways them, that plants generate their own cacophony of sounds is also well established in the literature (e.g., Milburn and Johnson 1966; Tyree and Sperry 1989; Kikuta et al. 1997; Laschimke et al. 2006; Gagliano, Mancuso, et al. 2012). The present review aims at opening the ground for a systematic exploration on the potential functional, ecological, and ultimately evolutionary significance of acoustic communication between plants.

## DO PLANTS PRODUCE SOUNDS, AND DO THEY “LISTEN”?

That plants produce sound waves has been known for some time. Specifically, plants emit sound waves at the lower end of the audio range within 10–240 Hz (audio acoustic emissions) as well as ultrasonic acoustic emissions (UAE) ranging from 20 to 300kHz. Over the last 45 years, these acoustic emissions (and particularly the UAE) have been measured and described several times (Milburn and Johnson 1966; Tyree and Sperry 1989; Kikuta et al. 1997; Laschimke et al. 2006). Acoustic emissions are generally interpreted as the result of the abrupt release of tension in the water-transport system of plants following cavitation as water is pulled by transpiration from the roots through the xylem to the leaves (see Cohesion Theory, Dixon and Joly 1895; but also Zimmerman 1983). Cavitation occurs when dissolved air within the water expands in the xylem conduits, eventually generating air bubbles (embolism), occluding the conduits and making them unavailable to transport water (reviewed by Tyree and Sperry 1989). In this context, these acoustic signals are simply emitted as an incidental by-product of the physiological/biomechanical process of cavitation and in fact, many authors have conveniently used them as an indicator of cavitation, particularly in drought-stressed plants (Peña and Grace 1986; Raschi et al. 1989; Jackson and Grace 1996; Qiu et al. 2002; Perks et al. 2004; Rosner et al. 2006). Nonetheless, others have argued that these plant sounds are not caused by cavitation disruption of the stressed water column, but rather, that they are induced by a largely stable bubble system of the xylem conduits capable of transporting water in travelling peristaltic waves (Laschimke et al. 2006). Although it remains undisputed that cavitation can induce acoustic emissions, the acoustic signals emitted by plants are so numerous that it always seemed extremely unlikely that each acoustic event was attributable to cavitation alone (Raschi et al. 1990; Zimmermann et al. 2004; Laschimke et al. 2006) and in fact, recent evidence now indicates that plants generate sounds independently of dehydration and cavitation-related processes (Gagliano, Mancuso, et al. 2012).

**Figure 1:**
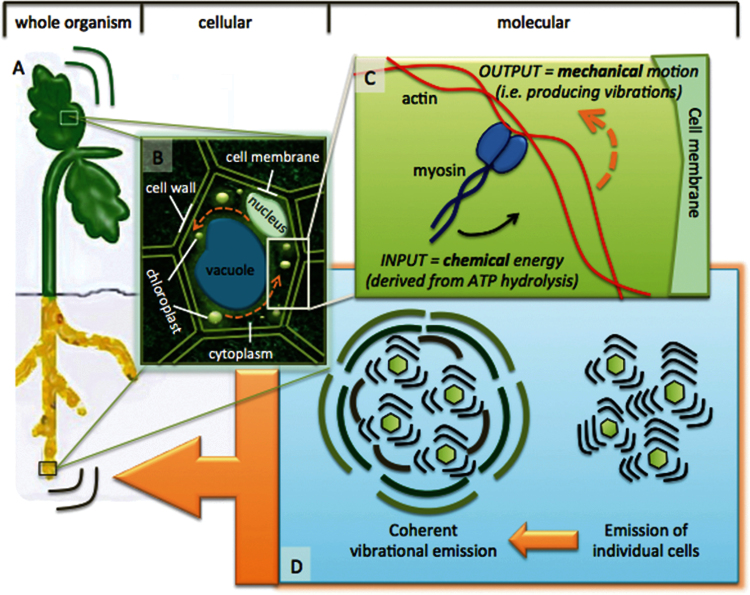
A putative model of a mechanism underlying sound production in plants. Plant cells vibrate as a result of the active movement of organelles within the cell (i.e., cytoplasmic streaming; orange arrows in panel B). Cytoplasmic streaming is caused by the activity of motor protein myosins as they slide along actin filaments using the hydrolysis energy of adenosine triphosphate (panel C). As the nanomechanical motion generated within a cell reflects the unique metabolic status of that cell, this information is contained in the emitted vibrational wave. Vibrations from individual cells propagate through the medium as sound waves and reach neighboring cells; if the receiving cells are receptive to that particular frequency, they will also start vibrating proportionally to the intensity of the received signal and when all the cells are “in tune,” the signal is amplified (panel D). As the signal extends outside the leaf or root of the plant, it conveys information about that plant to neighboring plants or other organism (panel A).

The mechanics of how plants produce sounds are still unknown. Plants are unlikely to possess the specialized morphological structures and/or organs that animals have evolved to produce sound; nonetheless, the biophysical principles at the cellular and molecular level may not be so dissimilar and in this context, the fundamental mechanism of sound production across all eukaryotes may be highly conserved. I propose here a putative model to start examining this phenomenon. Initially, we need to consider that sound waves are generated by objects that vibrate and in all eukaryotes, cells and their components vibrate as a result of intracellular motions generated by cellular processes such as the activity of motor proteins and the cytoskeleton (Howard 2009; Figure 1B, cytoplasmic streaming represented by the orange arrows). Specifically, motor proteins such as myosins, a family of mechanochemical enzymes, use chemical energy derived from the hydrolysis of adenosine triphosphate in actin filaments to generate mechanical motion and hence vibrations (Figure 1C). Using atomic force microscopes, such nanomechanical motions have been measured in different systems from vertebrate cardiomyocytes (i.e., heart cells, Domke et al. 1999) and auditory hair cells (i.e., spontaneous oscillations that play a role in active amplification of weak sounds in hearing, Jülicher 2001) to tiny microbial cells (e.g., the baker’s yeast, *Saccharomyces cerevisiae*, with motions in the order of 0.8–1.6kHz; Pelling et al. 2004). Because cells are imbedded in a tissue and hence surrounded by other cells, individual cells are affected by the mechanical property of neighboring ones and this eventually builds up into a collective mode (i.e., coherent excitation, see Pokorný 1999; Figure 1D) and results in the amplification of the signal. In plants, the radiated power of numerous cells working in such a concerted way has been theoretically predicted to be sufficient for observable effects, leading to acoustic flows in the order of 150–200kHz (Perelman and Rubinstein 2006). If such mechanical vibrations or sound waves can extend over large distances within the organism and also outside the organism (Figure 1D to A), then the possibility arises that plants may actually use sound to communicate with other plants or organisms.

Whichever explanation for the origin of acoustic emissions from plants is correct, the fact remains that plants emit sounds and they “hear” them too. Indeed, besides the folkloristic and at times esoteric reports of the influence of sound, and more specifically music, on plants (Backster 1968; Tompkins and Bird 1973), decades of scientific research indicate that plants do respond to sound waves of different frequencies by modifying germination and growth rates (Klein and Edsall 1965; Weinberger and Burton 1981; Takahashi et al. 1992). Moreover, sound waves elicit changes at the molecular and physiological level, including the levels of polyamines (Qin et al. 2003) and important phytohormones (e.g., indole acetic acid and abscisic acid; Wang et al. 2004), the regulation of antioxidant enzymes (Li et al. 2008), the uptake of oxygen (Qin et al. 2003), the synthesis of RNA and soluble proteins (Yi et al. 2003; Wang et al. 2003), and perhaps most importantly, gene expression (Jeong et al. 2004). The specific sensory mechanisms available in plants for detecting sound are still unclear although they are likely to be an aspect of the multifaceted phenomenon of mechanosensing, the intrinsic ability to sense and respond to mechanical perturbations, which all organisms exhibit in order to grow and develop. In fact, a wide diversity of mechanosensitive (MS) channels is present in a broad range of organisms from single-celled bacteria to complex multicellular animals and plants (reviewed by Haswell et al. 2011). Although wide structural diversity seems to have evolved to accommodate the physiological necessity of detecting forces of significantly different magnitudes (Sukharev and Anishkin 2004), MS channels in different organisms may nonetheless serve similar functions. In animals, for example, some MS channels are implicated in the perception of mechanical stimuli such as sound and touch (reviewed by Arnadóttir and Chalfie 2010); available evidence suggests that MS channels linked to changes in ion fluxes (primarily in cytostolic Ca^2+^) may play a similar function in plants (Haswell et al. 2008; review by Monshausen and Gilroy 2009) and hence, this may be a fruitful starting point for investigating the response pathways triggered by acoustic stimulations.

Clearly, our understanding of how plants produce and respond to sound is still noticeably limited and requires further detailed investigation. Yet, if elucidating the mechanisms by which plants produce and perceive acoustic signals is a major challenge for future research, we know even less about how plants may use them and what potential ecological role sound plays in a plant’s life.

## WHY SHOULD PLANTS EMIT SOUND, AND DO THEY MEAN ANYTHING?

Physical signals such as sound propagate rapidly, conveying real-time information. Moreover, acoustic signals can be altered in important ways to deliver instantaneous changes in the signal emitted; such signals can be analyzed quickly, sensed at very low intensity and long distances. Most importantly, acoustic signals can be generated and transmitted at a relatively low energy investment because they use energy liberated from biophysical processes (e.g., birds, Ward et al. 2004; reptiles, Conley and Lindstedt 1996; bacteria, Reguera 2011). Because of its very nature, sound can therefore offer an effective mechanism for signaling when a rapid and instantaneous response is required (e.g., much faster and possibly energetically cheaper than the chemical signaling, given the high metabolic costs associated with the synthesis of the volatile and allelochemical messengers commonly used by plants for such purpose; Paré and Tumlinson 1999). Yet, the role and potential adaptive utility of sound in plant communication remains hitherto unexplored. This issue can be tackled from a physiological viewpoint by considering that plants detect and deal with competitors and consumers through a very sophisticated sensing network mediated by phytohormones, which initiate responses to neighbors or canopy shade (i.e., shade avoidance syndrome) and chemical defenses to herbivore damage. These hormones may be a potential point of interaction or “cross-talk” forming a complex web of overlapping pathways that affects the mechanisms involved in competition and defense signaling, including mechanoreception of pressure waves (i.e., sounds). For example, indole acetic acid is known to play an important role in modulating defense responses induced by wounding (Fabbri et al. 2000) as well as initiating the multiple changes in body plan, such as stem elongation associated with shade avoidance (Tao et al. 2008; Ross et al. 2011). Interestingly, this same hormone is also implicated in the mechanisms that mediate sound-induced morphological changes of the callus (Wang et al. 2004), required for facilitating rapid cell proliferation in wounded tissues. Moreover, by concurrently inducing a decrease in the levels of abscisic acid, which normally inhibits stem elongation (Hasson and Poljakoff-Mayber 1983), it may be speculated that the resulting sound-induced morphological responses also facilitate above-ground competitive ability.

Because no scientific studies that I am aware of have been conducted on the potential acoustic communicative abilities of plants, one way to present a critical discussion of the topic is through a comparative approach using studies of acoustically, and in particular ultrasonically communicating taxa. These studies are selected examples primarily including terrestrial vertebrate animals because their ability to use acoustic (and specifically ultrasonic) communication is widespread although it is by no means the exclusive prerogative of this group. Indeed, we know that marine vertebrates from the largest whales to centimeter-sized fish engage in as much acoustic communication as their terrestrial counterparts (Myberg 1997; Bass and McKibben 2003) and many invertebrate animals from ultrasonic whispering moths in courtship (Nakano et al. 2009) to sessile species, which go about their lives firmly attached to the substrate are able to detect and respond to acoustic signals (Vermeij et al. 2010). Moreover, if we recall that even bacteria have the ability to communicate using ultrasonic sound waves (Matsuhashi et al. 1998), the idea that plants may communicate via sound signals should no longer be perceived as a research oddity. In the following paragraphs, I will offer 2 analogies as examples illustrating why this may be an oversight in our understanding of the sensory and communicative complexity of plants.

### Example 1. Ultrasonic utterances as the mere by-product of physiological processes

Infant rodents respond to extreme cold exposure with bradycardia, behavioral arousal, and production of ultrasounds (Blumberg et al. 1999). Just like sneezing, coughing, and wheezing suggest physical ailments in humans, the emission of ultrasounds by infant rodents seems not to be driven by any motivation to communicate acoustically or not even to emit sounds, but rather the result of a reflexive physiological/biomechanical process (i.e., the abdominal compression reaction) that produces sound as a by-product (Blumberg et al. 2000). Specifically, extreme cold exposure that may occur during isolation from the nest entails pronounced decreases in cardiac rate; by constricting the larynx during expiration and thus contributing to increase in intra-abdominal pressure (which results in involuntary ultrasonic emissions), rodent pups are able to propel blood back to the heart and maintain cardiac output while physiologically challenged (Kirby and Blumberg 1998). It is tempting to suggest that the abdominal compression reaction process by which cold-stressed pups inadvertently emit ultrasounds is analogous to the cavitation process described for drought-stressed plants. In both cases, the acoustic emissions seem to be just the mere and simple by-product of physiological and biomechanical strain. Yet, the incidental nature of ultrasonic emissions does not preclude the evolutionary development of a communicatory relationship between individuals. Do acoustic emissions from one plant affect the behavior of the surrounding plants? In the rodent example, the ultrasonic vocalizations of a pup being cooled outside the nest reliably elicit a phonotaxic response in the mother (Ehret and Haack 1984). Hence, regardless of the proximate cause of the signal’s emission, these ultrasounds trigger a behavior in the mother that is beneficial to the signaling pup (Blumberg and Alberts 1997). Clearly, this is a signal that transfers some information to the receiver, whose behavioral activities have changed in an adaptive way, hence increasing the genetic fitness of the pup–mother system. Accordingly, although the term of “true communication” is generally reserved to the intentional transfer of signals that benefits both sender and receiver (Bradbury and Vehrencamp 1998), the basis for a communication system to be established may not necessarily require intention or benefit for all parties involved. By studying whether the acoustic cues have a function and if so, how do these sounds affect responses in other organisms, plants, and animals alike (see Box 1), we can pursue questions about plant communication and test the potential role and adaptive benefits of acoustic emissions in plant assemblages.

BOX 1. **EXTRACTING AND USING INFORMATION**

The study of the mechanisms organisms use for extracting information from their environment and how they use this information for communication is an issue that has attracted lots of attention. Unfortunately, the task of unraveling these mechanisms and processes has proved to be a challenging endeavor in animals, let alone in plants. Accordingly, a different approach may be required and we could start from considering that communication is not always the final accomplishment. A lot of information only travels one way and this is sufficient to make a living. This approach is recognized broadly as *sensory ecology*, sometimes entering the fruitful and fascinating realm of *physical ecology*. The first is an interdisciplinary field dealing with primarily mechanistic questions of how organisms acquire and respond to information, whereas the latter examines more functional questions of what kind of information is obtained, and why the information is useful to the organism within a physical perspective. Because these relatively new approaches combine behavioral, physical, chemical, physiological, and evolutionary issues, both may be able to offer some inspiration and real guidance for how the investigations into plant sound emission and perception should proceed.

### Example 2. Alarm calling

In the presence of a predation threat, animals show a wide variety of responses, which generally include the combined use of visual, auditory, or olfactory signals to communicate to conspecifics or to predators (Caro 2005). Particularly, alarm calling has evolved as a key antipredator strategy in a wide range of species because auditory signaling is a very efficient way of communicating a substantial amount of information rapidly. Acoustic signals can be encoded across multiple channels (many natural sounds are in fact complex and broadband), are not limited by light availability, travel well even around corners, and require no visual contact or immediate proximity between signaler and receiver. Like animals, plants have an armory of signals that reach the open air in response to encounters with enemies. And over the last few decades, we have begun to appreciate that plants can warn each other of approaching insect attacks using an extensive vocabulary of chemical molecules, such as herbivore-induced volatile organic compounds. Through this airborne plant–plant communication channel, plants are able to respond to cues produced by injured neighbors when they are not yet attacked or damaged themselves, hence allowing for preemptive defensive responses (Paré and Tumlinson 1999; Karban et al. 2000; Heil and Ton 2008). Still, plant defenses are often assumed to be primarily limited to chemical traits that do not involve other modes of communication and we are yet to explore the possibility of plants using physical signals such as sound as part of their array of resources. This is surprising if we consider that acoustic signaling is particularly convenient when a swift and immediate response is required; in fact, sound propagates faster and allows for the (virtually) instantaneous transmission of a higher rate of information as well as a more accurate source localization (i.e., acoustic signals may incorporate features that degrade predictably with range allowing a receiver to estimate the signaler’s distance; Wiley and Richards 1978) than afforded by the dispersal of chemical signals (Walker 1998). In fact, because the dispersal of chemicals through the environment is contingent to molecular diffusion and bulk flow (e.g., wind direction), these signals are limited by a relatively low rate of information that they can carry and are often delivered with a variable delay (albeit the information they carry persist in the environment for far longer than acoustic signals). Additionally even accounting for varying threshold among taxa, acoustic signaling is generally not constrained by intensities as sounds can be sensed at very low intensities, whereas chemical signals often need to be produced in sufficiently high quantities to be detected by the cognate receptors after accounting for diffusion and dilution. And as previously mentioned, acoustic signals are likely to be energetically cheaper than chemical signals, which require considerable energy expenditure for the synthesis of the signal itself as well as its cognate receptor.

The idea that plant acoustic emissions may serve as short-range deterrents (or attractants) for some insects is not new as it had been proposed by Mattson and Haack (1987) and again very recently by Dunn and Crutchfield (2009), who speculated that these emissions influence the behavior of such insects as wood borers. Taking advantage of the rapid attenuation of the ultrasonic component of their acoustic emissions, could plants be broadcasting warning calls to their close neighbors without alerting the attacking herbivore? Given the increased portability of detection devices and microphone arrays, I argue that studies that explore these questions would be both highly feasible and timely.

## CONCLUDING REMARKS

For over 100 years, scientists disbelieved the data that showed that bats could orient using sound; it was this scientific disbelief that hampered the discovery of laryngeal echolocation in these animals (Teeling 2009). Today, not only the use of ultrasounds by bats epitomizes the concept of echolocation and biosonar in nature, but further investigation using playback experiments has also demonstrated the utility of such ultrasonic signals as social calls for bats to communicate with conspecifics (Jones 2008). The current lack of studies on plants and sound prevents drawing any firm conclusions on the potential bioacoustics abilities of these organisms at this stage (but see Gagliano, Mancuso, et al. 2012; Gagliano, Renton, et al. 2012).

We should expect the answers to the questions raised here and more (see Box 2) to emerge at the interface between disciplines, namely ecology and acoustics, because similar joint ventures have been previously shown to be particularly fruitful. The birth of plant chemical ecology, for example, unveiled the strikingly “talkative” nature of plants and the eloquence of their volatile vocabulary. The partnership between ecology and chemistry greatly advanced our understanding of plants, and it may now serve as an inspiration of the kind of purposeful cooperation between disciplines that will most likely lead to a new appreciation for the acoustic world of plants.

BOX 2. **OUTSTANDING QUESTIONS AND FUTURE DIRECTIONS**

- Although it is reasonable to conceive that plants use sounds as a source of information, incidentally or deliberately, to enhance their survival, what are the best species and experimental systems for exploring these ideas?
- Both emission and detection of sound may have adaptive value in plants, but what lacks to date is solid evidence about both of these processes, but particularly reception. How do plants perceive sound? How do we go about identifying receptor mechanisms and studying their function?
- Although we now have some evidence that sound as a stimulus changes some aspects of plant behavior, physiology, or morphology, how should we test for its specificity? Are the effects of sound distinctly different from other type of disturbances such as gentle mechanical inputs?
- Given the paucity of reports in the literature on what sort of sounds can be present under natural conditions, how should we overcome the logistically challenging task of measuring the fitness of a plant in an environment with different sounds or acoustic transmission characteristics?

In conclusion, a considerable body of evidence emerging from contemporary research in the plant sciences is increasingly recognizing plants as highly sensitive organisms that perceive, assess, interact, and even facilitate each other by actively acquiring information from their environment (Karban 2008; Baluška 2009; Trewavas 2009). We now know, for example, that when attacked, plants “cry for help” by producing volatiles that attract carnivorous enemies of the attacking herbivores (Dicke 2009); and on the other hand, parasitic plants can recognize their prey at a distance and evaluate their nutritional value before deciding to invade them (Kelly 1992; Koch et al. 2004). Considering that these are only a very few examples of what plants do, the Aristotelian view of plants as automata-like passive and insensitive creatures seems to be no longer accurate. By relinquishing this out-of-date view of the plant world, I hope the ideas and questions presented here seduce the most enquiring aspect of our nature into exploring the world of plants in its full potential complexity.
